# Comparative transcriptomics reveals key regulatory networks underlying cold stress adaptation in oil palm (*Elaeis guineensis*)

**DOI:** 10.3389/fpls.2025.1687366

**Published:** 2025-11-26

**Authors:** Qiufei Wu, Xuanwen Yang, Qifeng Huang, Rui Li, Xianhai Zeng, Qihong Li, Zongming Li, Dengqiang Fu, Hongxing Cao, Xinyu Li, Xiaoyu Liu, Lixia Zhou

**Affiliations:** State Key Laboratory of Tropical Crop Breeding, Coconut Research Institute, Chinese Academy of Tropical Agricultural Sciences, Sanya/Wenchang, Hainan, China

**Keywords:** oil palm, cold stress, transcriptome, differentially expressed genes (DEGs), signaling pathway

## Abstract

**Introduction:**

Climate change has exacerbated cold stress, which severely impairs plant development. Oil palm (*Elaeis guineensis*), a tropical crop highly sensitive to low temperatures, exhibits stunted growth and yield reductions under such conditions.

**Methods:**

To investigate its cold stress response, oil palm seedlings were subjected to cold treatments, and their physiological and genetic adaptations were analyzed using fresh leaf samples. Key parameters, including antioxidant enzyme activity, reactive oxygen species (ROS) levels, photosynthetic pigment ratios, photosynthetic efficiency, and gene expression, were evaluated across exposure durations. Sequencing of the samples was performed using Illumina NovaSeq X Plus platform. Raw reads were processed using fastp (v0.18.0) to remove adapter-containing reads, exclude reads with >10% unidentified nucleotides (N), and eliminate reads where >50% of bases had Phred scores ≤20. The genome reference version is GCF_000442705.2 (https://www.ncbi.nlm.nih.gov/datasets/genome/GCF_000442705.2/).

**Results and discussion:**

Under cold stress, seedlings displayed a significant increase in superoxide dismutase (SOD, 546.08 U/g min FW) and peroxidase (POD, 153.27 U/g min FW) activities within 4 h compared with the control. Prolonged exposure (8 h) further elevated soluble sugar content (406.27 μg/g FW), malondialdehyde (MDA, 80.22 nmol/g), relative electrical conductivity (109.71%), and the carotenoid-to-chlorophyll ratio, indicating oxidative damage and membrane instability. RNA-seq analysis identified 144, 392, and 6,585 differentially expressed genes (DEGs) after 1, 4, and 8 h of cold exposure, respectively. KEGG pathway enrichment highlighted predominant associations with plant–pathogen interaction, plant hormone signal transduction, and the mitogen-activated protein kinase (MAPK) signaling pathway. Functional analysis revealed DEGs involved in four major hormone signaling pathways (auxin (AUX/IAA), jasmonic acid (JA), abscisic acid (ABA), and brassinosteroid (BR)), which also interact with the MAPK cascade to collectively regulate oil palm cold stress adaptation and growth adjustments. This study provides comprehensive insights into the genetic and molecular mechanisms underlying cold tolerance in oil palm, offering a basis for breeding cold-resistant cultivars.

## 1 Introduction

*Elaeis guineensis* Jacq., commonly referred to as oil palm, is a long-lived woody monocot in the Arecaceae family. Recognized as the most productive oil crop worldwide, it plays a major role in the global vegetable oil industry. Palm oil, extracted from its fruit, is the most widely consumed edible oil, contributing nearly 35% to total global vegetable oil production, which surpassed 70 million metric tons in 2023 (Abubakar and Ishak, 2024; VanderWilde et al., 2023). The oil palm industry forms a high-value agricultural supply chain encompassing cultivation, oil extraction, and downstream processing for applications in food, cosmetics, biofuels, and other sectors (Al-Madani et al., 2023; Yılmaz and Ağagündüz, 2022; Chaparro et al., 2023). Malaysia and Indonesia dominate global production, jointly contributing over 85% of palm oil output (Danylo et al., 2021). Beyond economic significance, oil palm plantations support livelihoods for millions across Southeast Asia, Africa, and Latin America (Lai et al., 2022). Currently, the domestic cultivation of oil palm in China has not yet developed into a large-scale industry, with a total planting area of approximately 667 hectares scattered across regions such as Hainan, Yunnan, Guangdong, Guangxi, and Fujian. Expanding oil palm cultivation could significantly increase China’s self-sufficiency rate in edible oils, thereby enhancing national food security. It would also improve the efficiency of land resource utilization, boost farmers’ incomes, and promote regional economic development. Moreover, the growth of the oil palm industry could drive progress in related sectors such as agricultural technology, seed breeding, oil processing, and bio-new energy applications (John Martin et al., 2022). In China, oil palm cultivation is limited to tropical regions, and commercial expansion has been constrained by climatic limitations (particularly cold sensitivity) and suboptimal yields, necessitating heavy reliance on imports (Sarkar et al., 2020; Zhou et al., 2022).

Cold stress significantly hinders crop growth, development, and productivity and induces complex physiological responses. It suppresses growth processes, impairing seed germination, seedling establishment, flowering, and fruit set, ultimately leading to reduced germination rates, stunted seedling growth, and diminished seed set (Shahan, 2020). These adverse effects are especially detrimental to crops in tropical and subtropical regions, often causing substantial yield losses and declines in quality (Matsukura et al., 2024). At the cellular level, cold stress promotes the excessive accumulation of ROS, including superoxide (O_2_^–^), hydrogen peroxide (H_2_O_2_), and hydroxyl radicals (•OH), which induce membrane lipid peroxidation and damage to proteins and DNA (Xiang et al., 2025). To counteract oxidative stress, plants activate antioxidant defenses involving key enzymes such as Superoxide Dismutase (SOD), peroxidase (POD), and catalase (CAT) (Amini et al., 2021). Notably, at low concentrations, ROS act as signaling molecules that activate pathways such as MAPK and calcium (Ca^2+^) signaling cascades to regulate cold-responsive gene expression (Zhang et al., 2023).

Cold temperatures also disrupt the balance between plant growth and stress tolerance by activating a complex hormonal signaling network. Key hormones such as ABA, BR, and auxin (IAA) play pivotal roles in regulating these adaptive responses (Waadt et al., 2022). This hormonal coordination facilitates defense mechanisms through interconnected ROS–hormone networks, including the ABA–ROS–CBF signaling axis. Deciphering cold signal perception and multi-hormone crosstalk is essential for uncovering novel targets to engineer stress-resilient crops (Li et al., 2022; Shi and Chan, 2014).

Transcriptome sequencing has emerged as a pivotal tool in plant science, providing comprehensive insights into gene expression dynamics across developmental stages, environmental stresses, and treatments (Liu et al., 2022). In oil palm, this technology has advanced genetic improvement by enabling the discovery of genes associated with fatty acid biosynthesis, cold stress responses, and complex metabolic pathways (Lei et al., 2014; Xiao et al., 2014; Li et al., 2020, 2022; Saand et al., 2022; Apriyanto and Ajambang, 2022; Lee et al., 2024);. Despite progress in high-throughput sequencing, the molecular mechanisms underlying cold adaptation remain incompletely characterized in oil palm, and the identification and functional analysis of cold-tolerance markers and pathways are still limited. This gap impedes molecular breeding for high-yielding, cold-resilient varieties. Here, we employ high-throughput transcriptome sequencing to dissect gene regulatory networks in oil palm under cold stress and identify key molecular components governing stress adaptation, with the goal of establishing a molecular framework for genetic enhancement of this crop.

## 2 Materials and methods

### 2.1 Experimental material and cold exposure

Six-month-old African oil palm (*Elaeis guineensis* var. pisifera) seedlings were obtained from Wenchang, Hainan Province, China (19.63° N, 110.94° E) and acclimatized under nursery conditions. Oil palm seeds were initially germinated in a growth chamber under controlled conditions: a constant temperature of 29°C, 85% relative humidity, and a 12-hour photoperiod. Following germination, the sprouts were moved to a pre-nursery stage and placed in small polyethylene pots filled with a sterile peat–perlite mixture (3:1 v/v). At the two-leaf stage, the seedlings were transplanted into larger bags (35 cm × 50 cm) in the main nursery. The nursery environment was maintained at an average temperature of 28–32°C under natural sunlight. Seedlings received daily watering and were fertilized every two weeks using a balanced N-P-K fertilizer (mass ratio: 15-15-15). The bags were arranged in a triangular pattern with 90 cm spacing between them to ensure sufficient air circulation and light exposure. Twenty-four uniformly healthy seedlings were randomly divided into two experimental groups (n = 12 per group): (1) a control group maintained at 26°C with a 16 h light/8 h dark photoperiod, and (2) a treatment group exposed to 8°C for 1, 4, or 8 h under identical light (250 µmol/m^2^ s) and relative humidity (72-75%) conditions. The plant growth chamber (Baowen YL1, Shanghai, China) is equipped with built-in fluorescent lamps, whose light intensity is monitored in real time using a quantum sensor and automatically adjusted by the control system through regulating the lamp output. The chamber also incorporates humidification and dehumidification systems, which dynamically modulate relative humidity based on real-time feedback from high-precision humidity sensors, maintaining it within the range of 72–75%. Cold stress was induced in oil palm seedlings by exposing them to an artificial climate chamber pre-set to 8°C. All treatments were done at the same time of day to control the circadian effects. Each group had three biological replicates (one seedlings per replicate). Immediately following treatment, spear leaves were collected from both control and cold-treated seedlings, flash-frozen in liquid nitrogen, and stored at –80°C for RNA isolation and transcriptomic analysis.

### 2.2 Physiological and biochemical analyses

To evaluate cold stress responses, leaf samples were collected from the treated and control plants at 0, 1, 4, and 8 h post-exposure. Antioxidant enzyme activities, including SOD and POD, were determined using commercial assay kits (Micro SOD/POD Activity Assay Kits, Sangon Biotech, Shanghai, China). Photosynthetic parameters, including chlorophyll a/b ratio, carotenoid/chlorophyll ratio, net photosynthetic rate (*Pn*), intercellular CO*_2_* concentration (*Ci*), and stomatal conductance (*Gs*), were measured according to established protocols (Wang et al., 2015; Porra, 2002). Stress-related metabolites were quantified as follows: soluble sugars (Teixeira et al., 2012), MDA (Micro MDA Assay Kit, Sangon Biotech), and relative electrical conductivity (REC) (He et al., 2018). To estimate leaf cell membrane damage, REC was measured using an electric conductivity meter EC215 (Hanna Instruments Romania Srl, Nusfalau, Romania) (Niu et al., 2016). REC (%) was calculated the following equation, REC (%) = (S1/S2) × 100, where S1 and S2 refer to electric conductivity of live leaf sections and boiled leaf sections, respectively.

### 2.3 RNA extraction and transcriptome analysis

Total RNA was extracted using TRIzol Reagent (Invitrogen, Carlsbad, CA, USA) according to the manufacturer’s protocol. RNA quality was evaluated by RNase-free agarose gel electrophoresis and quantified using an Agilent 2100 Bioanalyzer (Agilent Technologies, Palo Alto, CA, USA). Poly(A)+ mRNA was enriched using mRNA capture beads and fragmented at elevated temperature. First-strand cDNA was synthesized using reverse transcriptase, followed by second-strand synthesis with end repair and A-tailing. Adapters were ligated to the cDNA fragments, which were then size-selected using Hieff NGS DNA Selection Beads. The final library was prepared by PCR amplification and sequenced on the Illumina NovaSeq X Plus platform. Raw reads were processed using fastp (v0.18.0) to remove adapter-containing reads, exclude reads with >10% unidentified nucleotides (N), and eliminate reads where >50% of bases had Phred scores ≤20. HISAT2 was used before StringTie assembly. To validate the consistency and reproducibility among biological replicates, we conducted a principal component analysis (PCA) of all samples.

Transcript quantification used a reference-based approach, with mapped reads from each sample assembled via StringTie (v1.3.1). Expression levels were quantified as FPKM (Fragments Per Kilobase of transcript per Million mapped reads) using RSEM, enabling standardized comparison of transcript abundance across samples. The FPKM calculation is: FPKM = (10^6^ × C)/(N × L/10^3^). where C is the number of fragments aligned to gene i, N is the total number of fragments mapped to all reference genes, and L is the length of gene i (bp). Differential expression analysis used DESeq2. Transcripts meeting false discovery rate (FDR) < 0.05 and |log2 fold change| ≥ 1 (linear fold change ≥ 2) were classified as DEGs. Gene Ontology (GO) enrichment DEGs were mapped to GO terms (http://www.geneontology.org/), and enrichment was evaluated using the hypergeometric test with FDR correction (FDR ≤ 0.05).

### 2.4 KEGG pathway enrichment

KEGG enrichment used the same hypergeometric framework as GO, with FDR correction (FDR ≤ 0.05). Protein–protein interaction (PPI) analysis was conducted using STRING v12.0 (https://string-db.org/) with an interaction confidence cutoff ≥ 0.700.

### 2.5 Expression profiles of DEGs by qRT-PCR

Eight randomly selected DEGs associated with cold stress (primers in **Supplementary Table 1**) were validated by qRT-PCR. Total RNA was extracted using TRIzol (Invitrogen), and first-strand cDNA was synthesized from 1 µg of RNA using MightyScript First Strand cDNA Synthesis Master Mix (Sangon Biotech) with oligo(dT)18 primers. qRT-PCR was performed in 10 µL reactions using 2 × SYBR Green qPCR ProMix (low ROX) on a Mastercycler ep realplex4 system under the following conditions: 95°C for 5 s, 55°C for 15 s, and 68°C for 20 s, followed by a melt curve from 60°C to 95°C over 20 min. All reactions were run in triplicate at both biological and technical levels. The expression levels of genes were normalized to those of ELF, which was previously found to be a stable reference gene under abiotic stress (Xia et al., 2014). Gene expression changes were calculated using the 2^–ΔΔCt^ method (Livak et al., 2001), with final Ct values representing the means of nine measurements. Statistical significance (p < 0.05) under cold treatments was assessed by one-way ANOVA with LSD *post hoc* tests (SPSS).

## 3 Results

### 3.1 Cold stress alters physiological and biochemical parameters in oil palm

**Figure 1:**
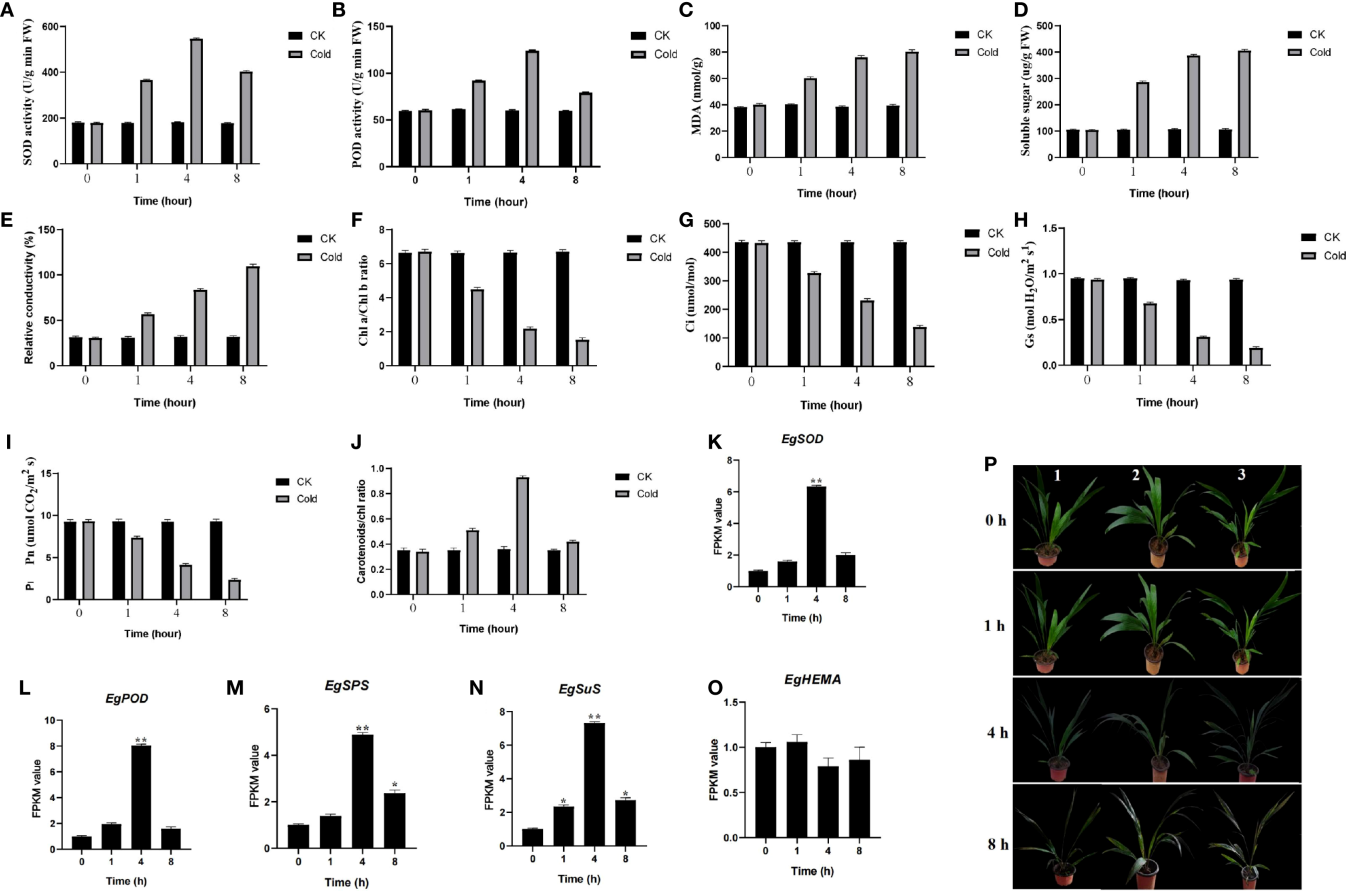
Effects of cold stress on the growth phenotype and physiological indices of oil palm seedlings. **(A–J)** Assessment of physiological and biochemical parameters in oil palm subjected to control and cold stress conditions at 0, 1, 4, and 8 hours of exposure. Data are presented as mean ± SD (n = 3). **(K–N)** FPKM value of *SOD, POD, SPS, SuS*, and *HEMA* genes under cold stress conditions. Asterisks represent significant difference at p ≤ 0.05(*) and p ≤ 0.01(**). **(P)** Growth phenotypes of seedlings from different treatment groups after the stress period. 1–3 means three biological replicates.

Plants were subjected to cold treatment for 0, 1, 4, and 8 h. Key physiological and biochemical parameters were analyzed, including antioxidant enzyme activities, ROS-related damage (MDA), soluble sugars, pigment content (chlorophyll and carotenoids), and gas exchange traits. SOD and POD activities increased during cold exposure, peaking at 4 h and decreasing by 8 h (**Figures 1A, B**). MDA content, soluble sugars, and REC increased progressively (**Figures 1C–E**). Conversely, chlorophyll content, *Ci*, *Gs*, and *Pn* declined (**Figures 1F–I**). Carotenoid/chlorophyll ratios were higher at 4 h relative to the control (**Figure 1J**). The phenotype of oil palm seedlings exhibited minimal change compared to the 0-hour baseline following one hour of cold stress. After four hours, however, brown spots emerged on the leaves, suggesting the onset of chilling injury. Within eight hours, brown spots had spread across nearly the entire leaf surface, indicating severe cold damage (**Figure 1P**).

### 3.2 Cold stress induces differential transcriptional reprogramming in oil palm

**Figure 2:**
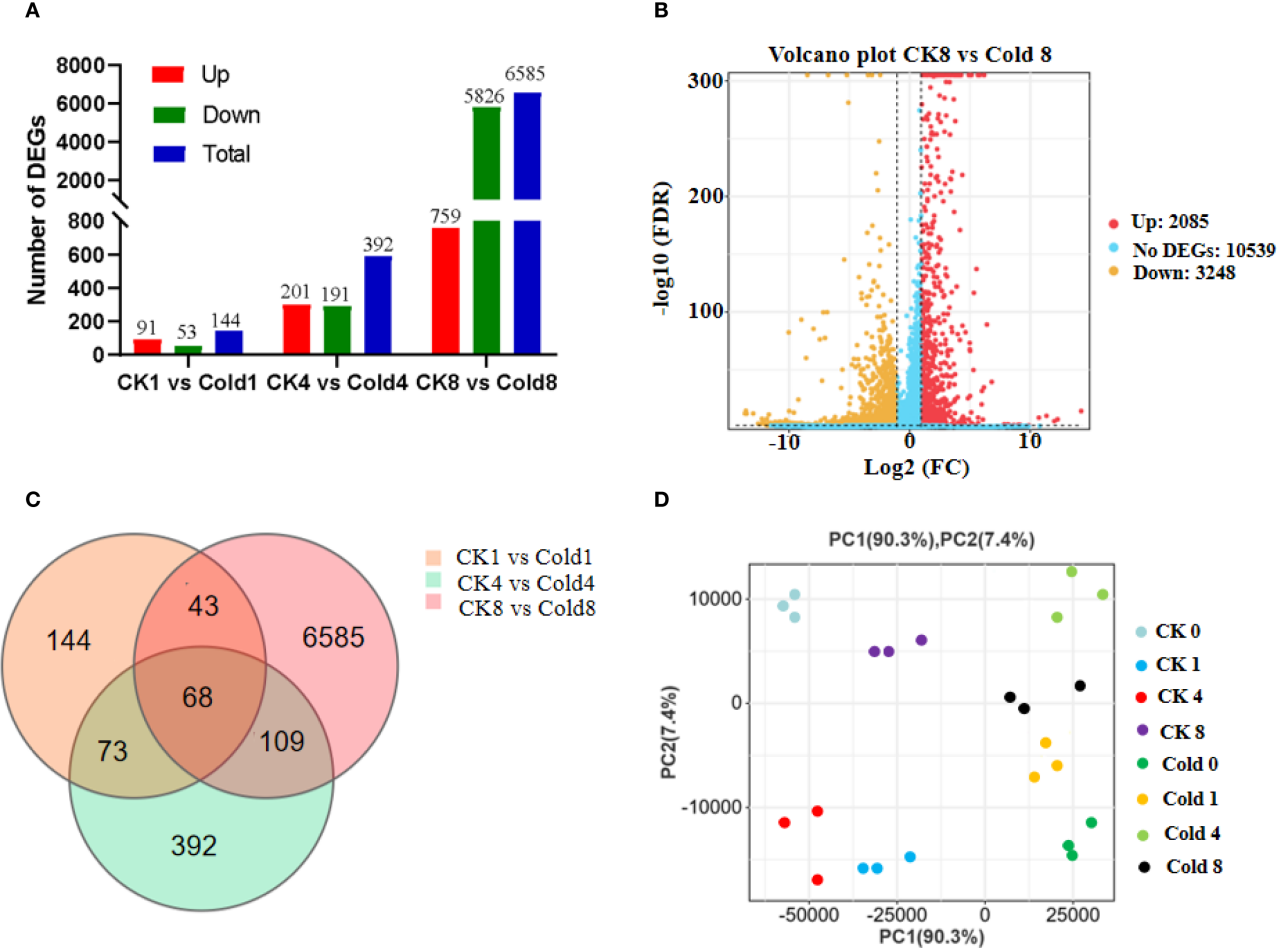
Transcriptional reprogramming in oil palm during cold stress. **(A)** Temporal dynamics of significantly upregulated (red) and downregulated (blue) differentially expressed genes (DEGs; FDR < 0.05, |fold change| ≥ 2). **(B)** Volcano plot of DEGs (8 h cold vs. control; red: upregulated, yellow: downregulated). **(C)** Venn diagram of DEG overlaps across time points; 68 genes consistently responded at all durations. **(D)** Principal Component Analysis (PCA) showing transcriptome separation by treatment time (PC1: 90.3%, PC2: 4.0% of variance).

Libraries generated 42.03–46.14 million high-quality paired-end reads (2 × 150 bp) per sample (~6.26 GB per replicate) with an average Q30 of 94.54% and a clean-reads percentage of 88.49% (**Supplementary Table 2**). The length of the DNA insertion fragment is 200–300 bp. The genome reference version is GCF_000442705.2 (https://www.ncbi.nlm.nih.gov/datasets/genome/GCF_000442705.2/). Employing thresholds of FDR < 0.05 and |fold change| ≥ 2, we identified DEGs across three pairwise comparisons (CK 1 h vs Cold 1 h, CK 4 h vs Cold 4 h, and CK 8 h vs Cold 8 h). The number of DEGs increased progressively with the duration of cold exposure, reaching a maximum of 6,585 DEGs in the CK 8 h vs Cold 8 h comparison (**Figures 2A, B**), reflecting a time-dependent expansion of transcriptional reprogramming. Venn analysis uncover both time-point-specific and shared DEGs, among which 68 were consistently regulated across all cold treatments, suggesting their role as potential core cold-response regulators (**Figure 2C**). Principal component analysis (PCA) showed clear separation among treatment groups, with PC1 and PC2 accounting for 90.3% and 4.0% of the total variance, respectively (**Figure 2D**).

### 3.3 Functional annotation of cold-responsive DEGs

**Figure 3:**
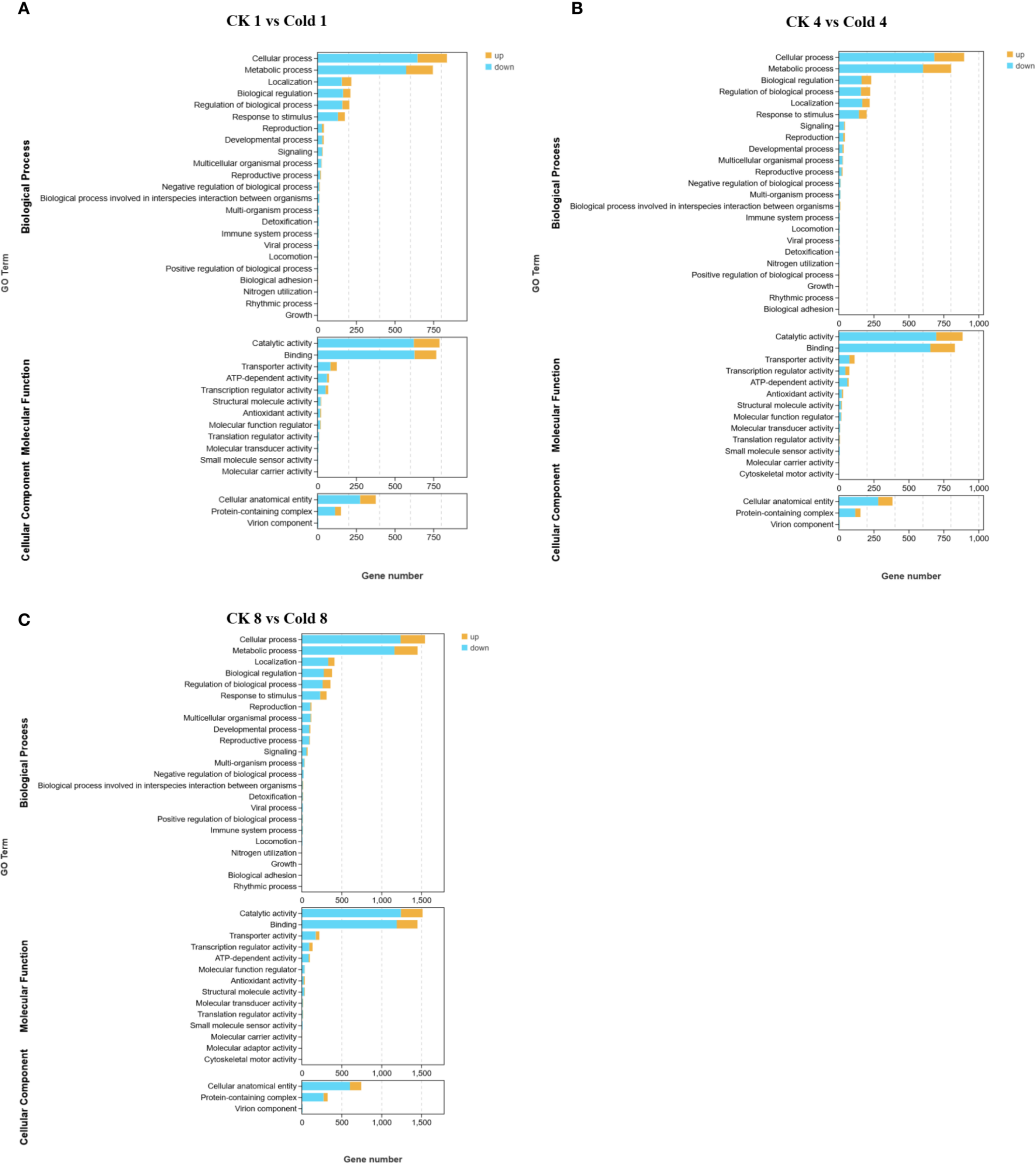
GO analysis analysis of DEGs. **(A)** ck 1 vs Cold 1, **(B)** ck 4 vs Cold 4, **(C)** ck 8 vs Cold 8.

**Figure 4:**
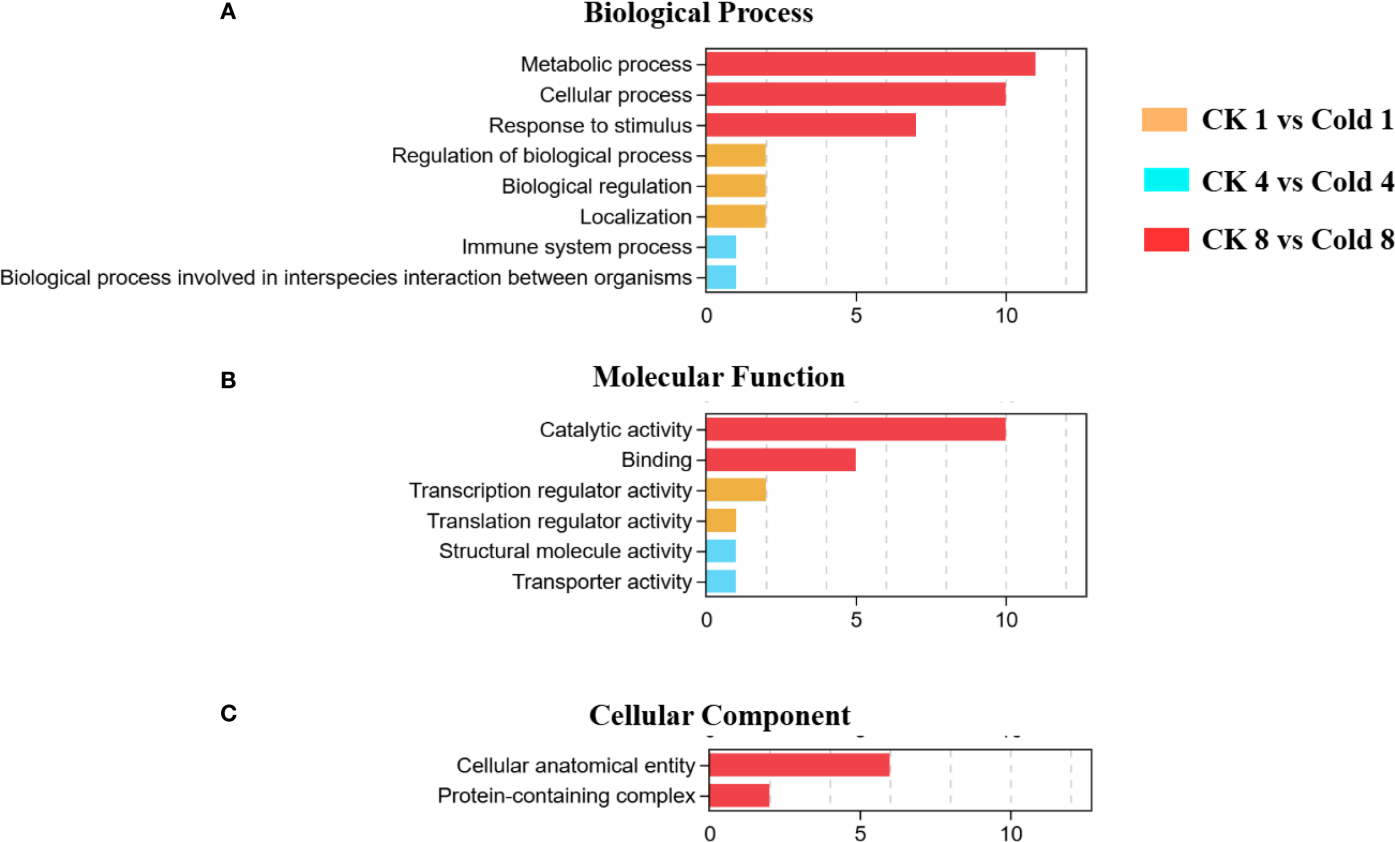
GO enrichment analysis of oil palm under cold stress. **(A)** Biological Process (BP), **(B)** Molecular Function (MF), and **(C)** Cellular Component (CC).

GO enrichment identified 35 significantly enriched terms (FDR < 0.05) at 8 h: 20 biological process (BP), 12 molecular function (MF), and 3 cellular component (CC) terms. Stress-response-related categories dominated BP, while oxidoreductase activity was prominent in MF. MF terms included binding, catalytic activity, transporter activity, transcription regulator activity, ATP-dependent activity, and antioxidant activity. BP terms included cellular processes, metabolic processes, biological regulation, response to stimulus, and localization. CC terms included cellular anatomical structures and protein-containing complexes (**Figures 3A–C**) (**Supplementary Table 3**). These results suggest coordinated adjustments at biological, cellular, and molecular levels (**Figure 4**). The comparison between CK 8 h and cold 8 h revealed significant biological characteristics. DEGs were primarily enriched in metabolic processes, cellular processes, and response to stimulus (**Figure 4A**), suggesting that cold stress may activate energy metabolism and secondary metabolite synthesis pathways as adaptive mechanisms. These DEGs likely participate in cell cycle regulation and cold stress responses, including oxidative damage mitigation and hormone-related signaling, highlighting cold stress’s disruptive effects on cellular equilibrium. In the MF category, most DEGs were strongly associated with catalytic activity and binding (**Figure 4B**), indicating potential alterations in enzymatic functions and molecular interactions during cold exposure. For the CC category, enriched terms were primarily related to cellular anatomical structures and protein complexes (**Figure 4C**), suggesting cold-induced modifications in cellular architecture and protein organization. Functional prediction and GO enrichment analysis underscored the critical involvement of diverse biological processes, molecular functions and cellular components in oil palm’s cold stress response. Notably, genes associated with metabolic pathways, enzymatic activities, and cellular structures emerged as key players in cold tolerance, likely contributing to cellular stability maintenance and enhanced stress resistance under cold conditions.

### 3.4 KEGG pathway enrichment and functional annotation

**Figure 5:**
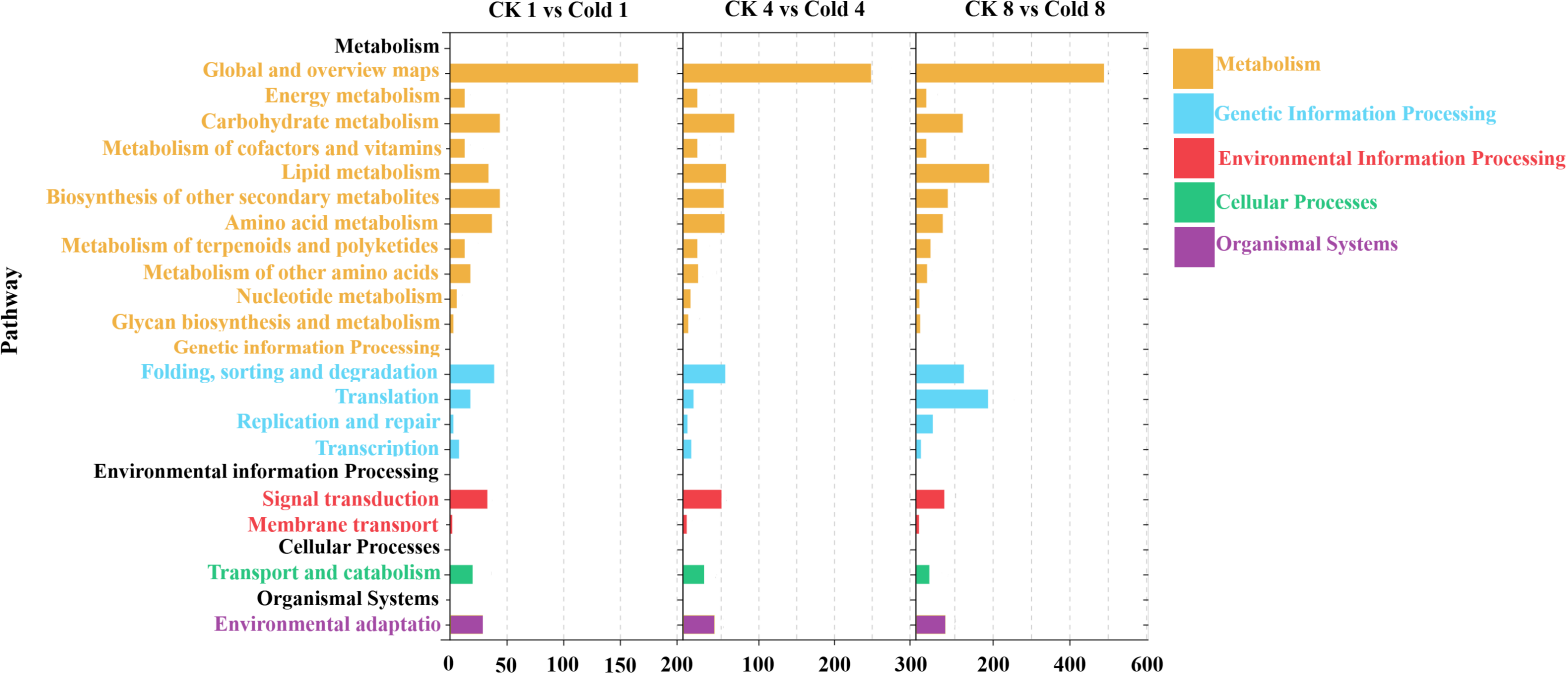
Comparative KEGG pathway enrichment analysis of DEGs in oil palm under cold stress was performed at 1 h (CK 1 h vs Cold 1 h), 4h (CK 4 h vs Cold 4 h), and 8 h (CK 8 h vs Cold 8 h).

Significantly enriched KEGG pathways (Q < 0.05) were classified into organismal systems, cellular processes, environmental information processing, genetic information processing, and metabolism (**Figure 5**), comprising 19 subcategories. Both up- and downregulated DEGs accumulated with prolonged cold exposure, with the highest numbers at 8 h (**Supplementary Table 4**).

**Figure 6:**
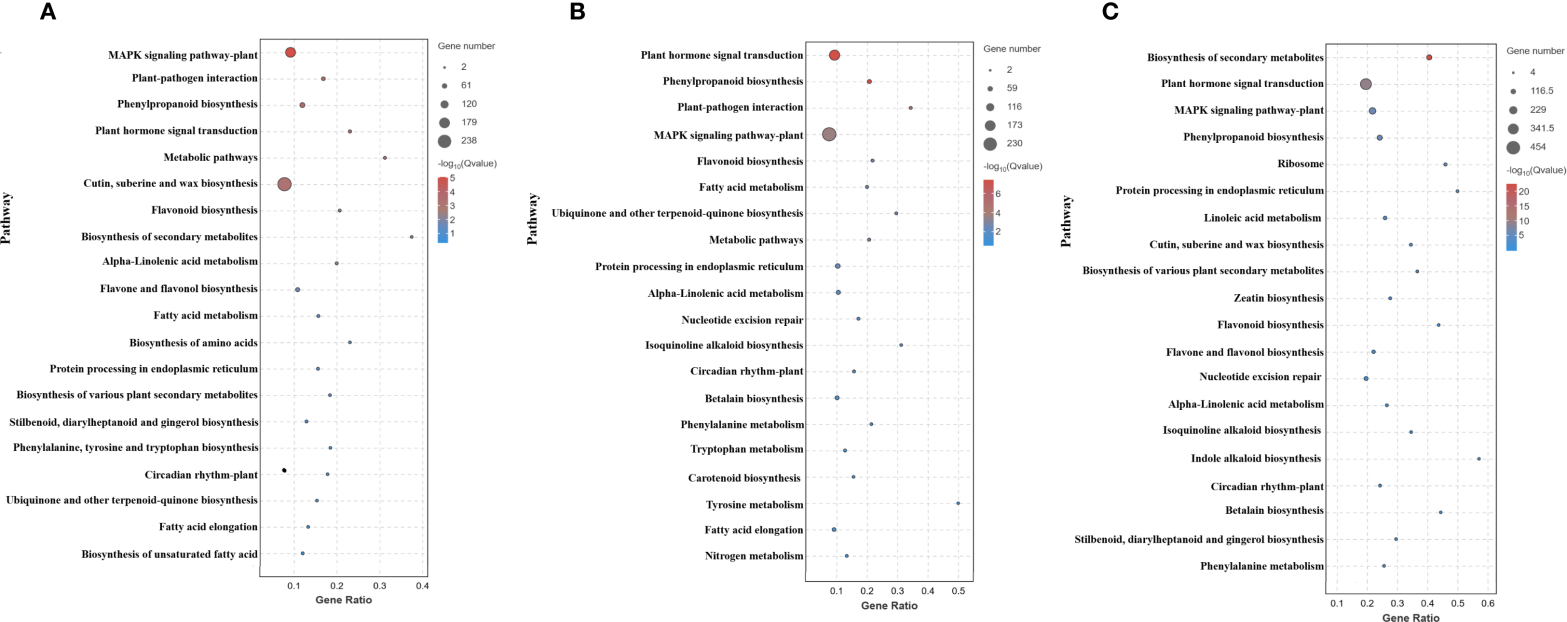
Top 20 enriched KEGG pathways: **(A)** 1 h, **(B)** 4 h, **(C)** 8 h cold stress. Each bubble represents a pathway, with its size indicating the number of enriched genes, while the color of the bubble reflects the significance level of the corresponding pathway.

Metabolic pathways dominated the transcriptome changes, including carbohydrate metabolism, secondary metabolite biosynthesis, and amino acid metabolism. Genetic information processing showed predominant upregulation. Environmental information processing, especially plant hormone signaling, the MAPK pathway, the phosphatidylinositol signaling system, and ABC transporters, was consistently downregulated. Cellular processes displayed initial downregulation (1 h) followed by increased upregulation (4–8 h), whereas organismal systems (environmental adaptation) were consistently downregulated (**Figure 5**). Across all times, the top 20 enriched pathways included MAPK signaling, plant–pathogen interaction, and phenylpropanoid biosynthesis (**Figures 6A–C**); secondary metabolite biosynthesis showed minimal expression changes at 8 h.

### 3.5 Cold-induced DEGs in plant hormone signaling

**Figure 7:**
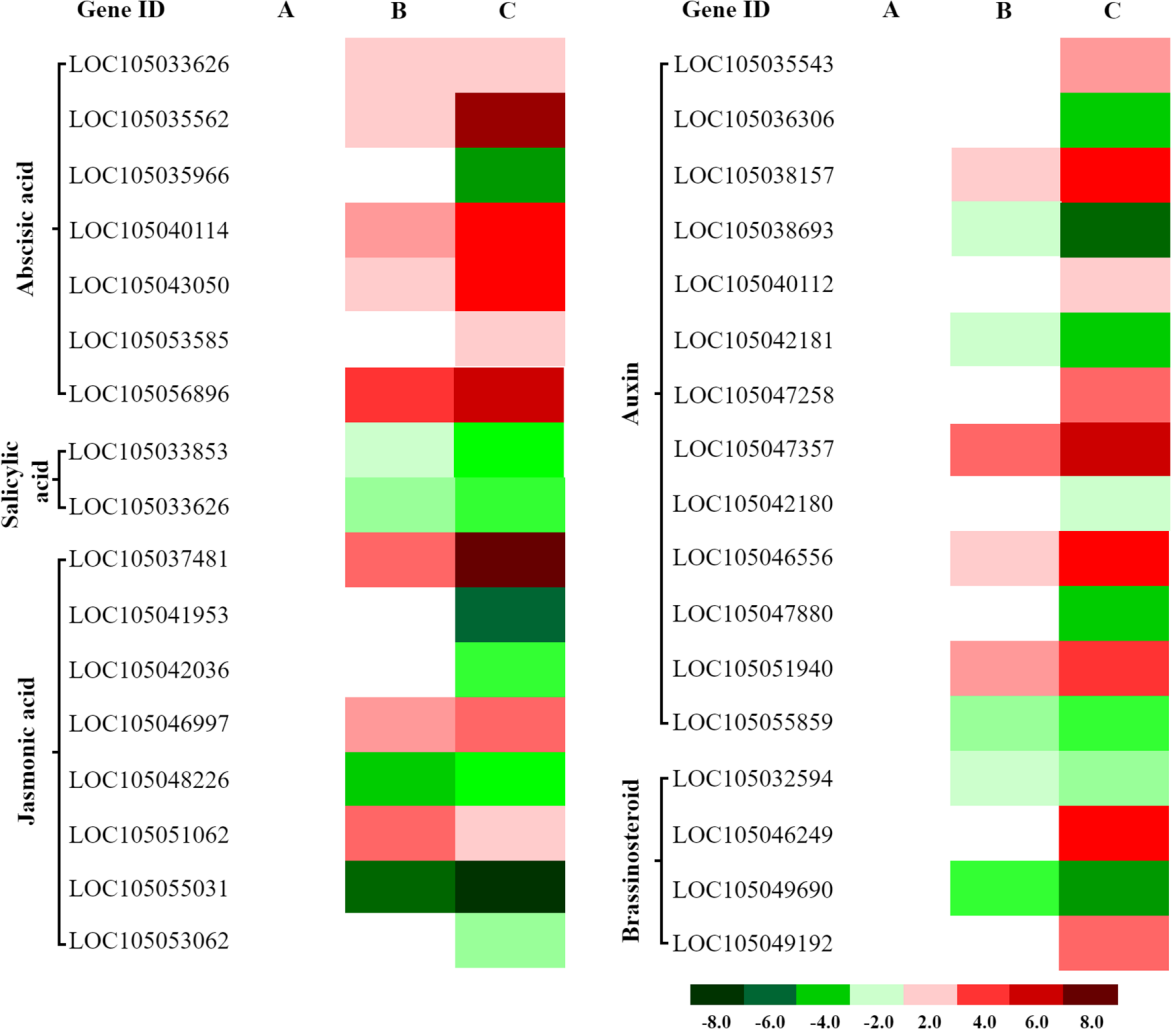
Differential expression of hormone signaling-related genes in oil palm under cold stress. Heatmaps display expression patterns of differentially expressed genes (DEGs) associated with abscisic acid (ABA), salicylic acid (SA), jasmonic acid (JA), auxin, and brassinosteroid (BR) signaling pathways at three time points: **(A)** 1 hour (CK 1 h vs. Cold 1 h), **(B)** 4 hours (CK 4 h vs. Cold 4 h), and **(C)** 8 hours (CK 8 h vs. Cold 8 h) of cold exposure. Red: Upregulated genes (log2FC > 1, FDR < 0.05). Green: Downregulated genes (log2FC < −1, FDR < 0.05).

Cold stress significantly altered expression of 45 genes in hormone signaling pathways (ABA, JA, auxin, BR, and SA) (**Supplementary Table 5**). The ABA pathway showed the strongest response (11 upregulated, 7 downregulated); auxin followed (8 up, 5 down); JA displayed moderate changes (3 up, 5 down); BR showed balanced regulation (2 up, 2 down); SA was minimally affected (1 up, 1 down). Overall, ABA, auxin, and JA pathways contained the most downregulated genes, with ABA also showing the greatest upregulation (**Figure 7**). Expression peaked at 4 h for representative proteins, whereas the largest number of DEGs occurred at 8 h, suggesting early regulation may involve post-translational mechanisms and later responses rely more on transcriptional modulation.

Cold stress initially induces a large-scale transcriptional suppression in oil palm seedlings, suggesting a conservative “niche adaptation” strategy that reduces overall metabolic activity to conserve energy and promote survival, rather than broadly activating defense pathways. This study reveals that low-temperature stress triggers a complex and coordinated reprogramming of hormone signaling. The central adaptive mechanism involves an “energy-saving defense” mode, rather than a global upregulation of gene expression. During later stress stages (8 h), transcriptomic reprogramming is characterized by a widespread downregulation of multiple signaling pathways, including auxin and jasmonic acid, likely to minimize metabolic expenditure and extend survival time. Conversely, the ABA pathway is specifically and strongly activated under low temperatures, functioning as a core regulatory hub for cold defense and mediating physiological responses such as stomatal closure. This marked contrast between the suppression of growth-related pathways (e.g., auxin, JA) and the activation of defense-related ABA signaling underscores a strategic ecological trade-off, sacrificing growth to ensure survival. The cold response also displays a temporal hierarchy, with early-stage (4 h) mechanisms relying on rapid post-translational protein modifications, and later-stage (8 h) adaptations driven by systematic transcriptional changes. Collectively, these results demonstrate that cold stress predominantly suppresses transcriptomic expression in oil palm seedlings, rather than enhancing individual gene expression.

### 3.6 Cold-regulated genes in the MAPK pathway

**Figure 8:**
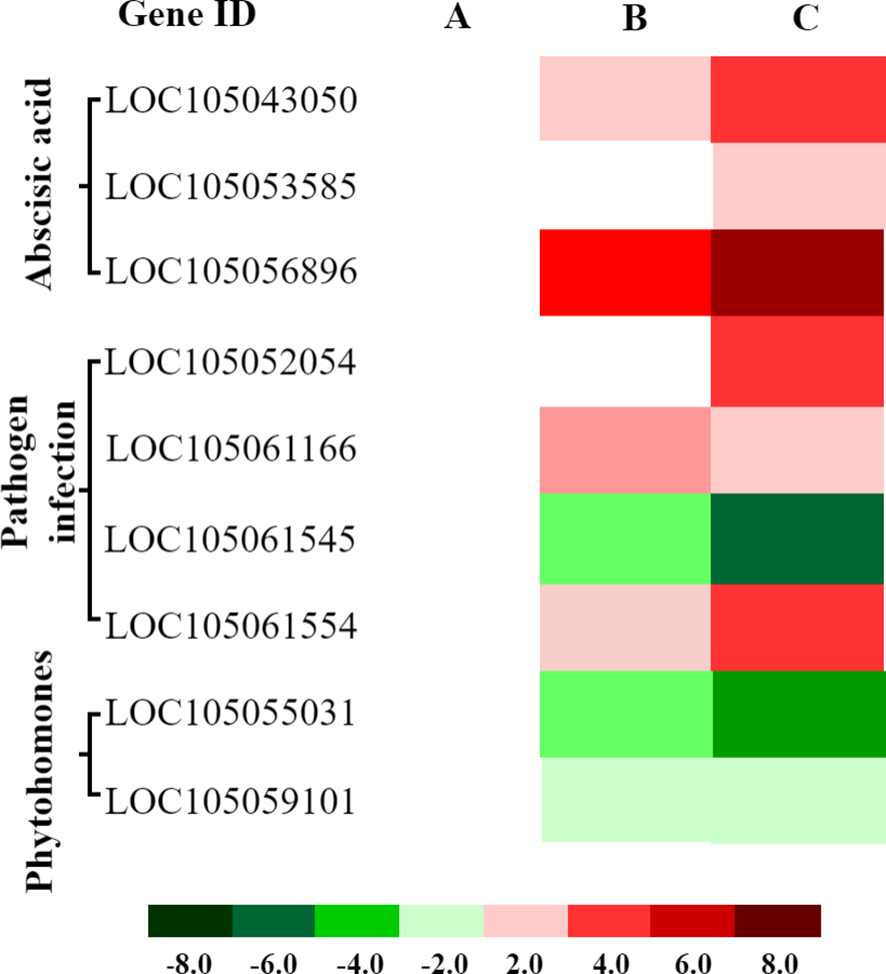
Expression patterns of cold-responsive DEGs in hormone signaling and stomatal development pathways mediated by MAPK cascade. Heatmaps display expression patterns of differentially expressed genes (DEGs) associated with ABA signaling, pathogen defense, and phytohormone signaling pathways at three time points: **(A)** 1 hour (CK 1 h vs. Cold 1 h), **(B)** 4 hours (CK 4 h vs. Cold 4 h), and **(C)** 8 hours (CK 8 h vs. Cold 8 h) of cold exposure. Red: Upregulated genes (log2FC > 1, FDR < 0.05). Green: Downregulated genes (log2FC < −1, FDR < 0.05).

Cold stress can induce the MAPK signaling cascade, which is involved in regulating cell division, differentiation, programmed cell death, and stress responses. We identified 15 cold-responsive DEGs associated with hormone signaling, stress defense, and pathogen resistance, including nine linked to ABA signaling (7 up, 2 down), four to plant–pathogen interaction (2 up, 2 down), and two to phytohormone signaling (both down) (**Figure 8**; **Supplementary Table 6**). These findings suggest that cold stress induces MAPK to coordinate downstream adaptive responses, These responses may include the enhancement of ABA-dependent stress resistance, the recruitment of pathogen defense mechanisms, and the suppression of certain hormone signaling pathways, potentially contributing to more efficient energy allocation.

### 3.7 Protein–protein interactions under cold stress

**Figure 9:**
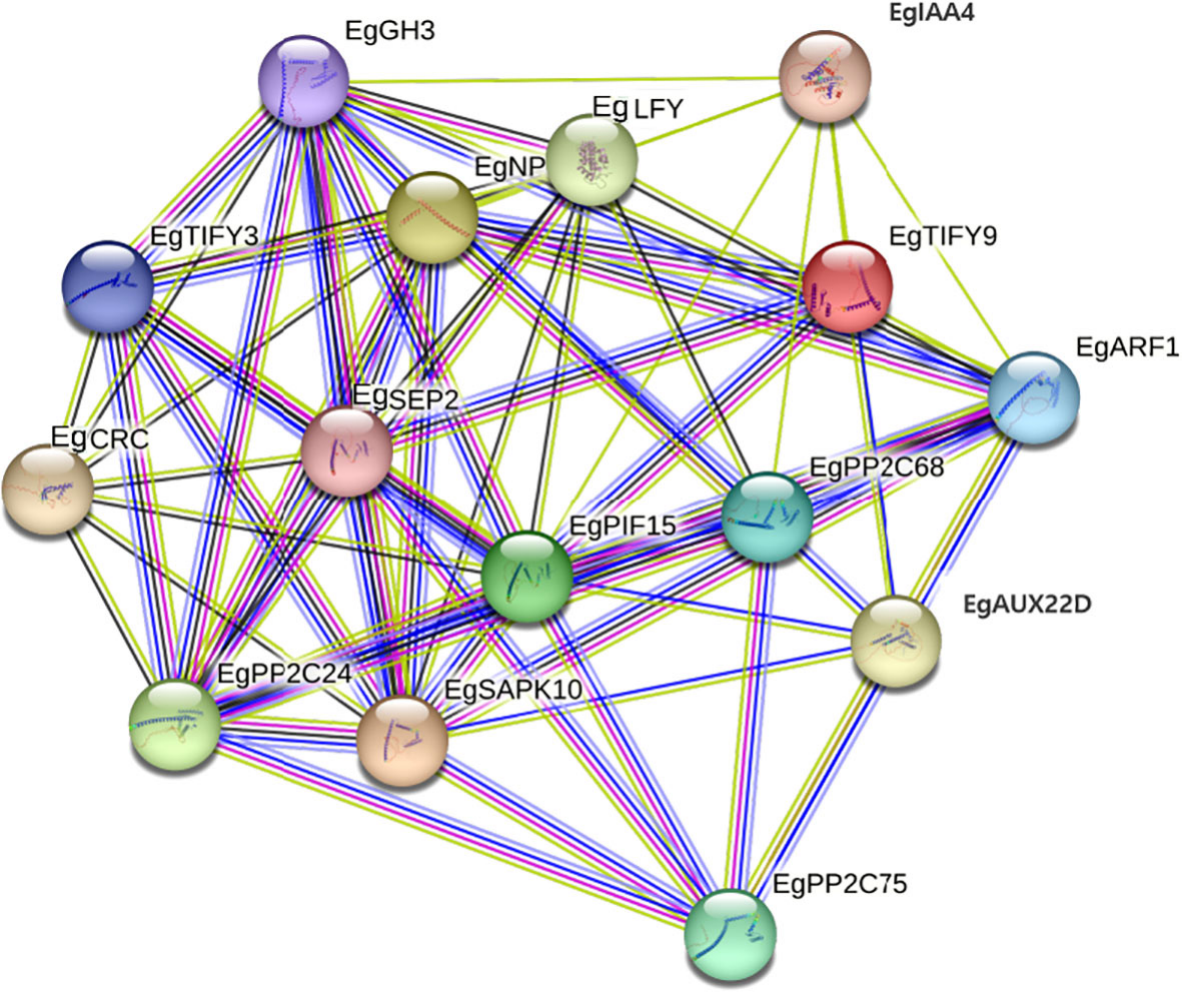
Analysis of protein-protein interaction (PPI) networks in oil palm under cold stress.

PPI analysis identified 15 core interacting proteins (EgIAA4, EgGH3, EgARF1, EgTIFY9, EgLFY, EgNP, EgTIFY3, EgCRC, EgSEP2, EgPP2C24/68/75, EgARF1, EgAUX22D, and EgSAPK10) constituting a potential cold-responsive regulatory module (**Figure 9**). The prevalence of PP2C family members highlights their roles in ABA signaling as negative regulators. The presence of auxin-associated proteins (EgIAA4, EgGH3, EgARF1) implies crosstalk between auxin and stress-responsive pathways (e.g., EgSAPK10). Collectively, these proteins may form an integrated network that coordinates hormonal and stress responses, with EgARF1 and PP2Cs as potential key nodes.

### 3.8 qRT-PCR validation

**Figure 10:**
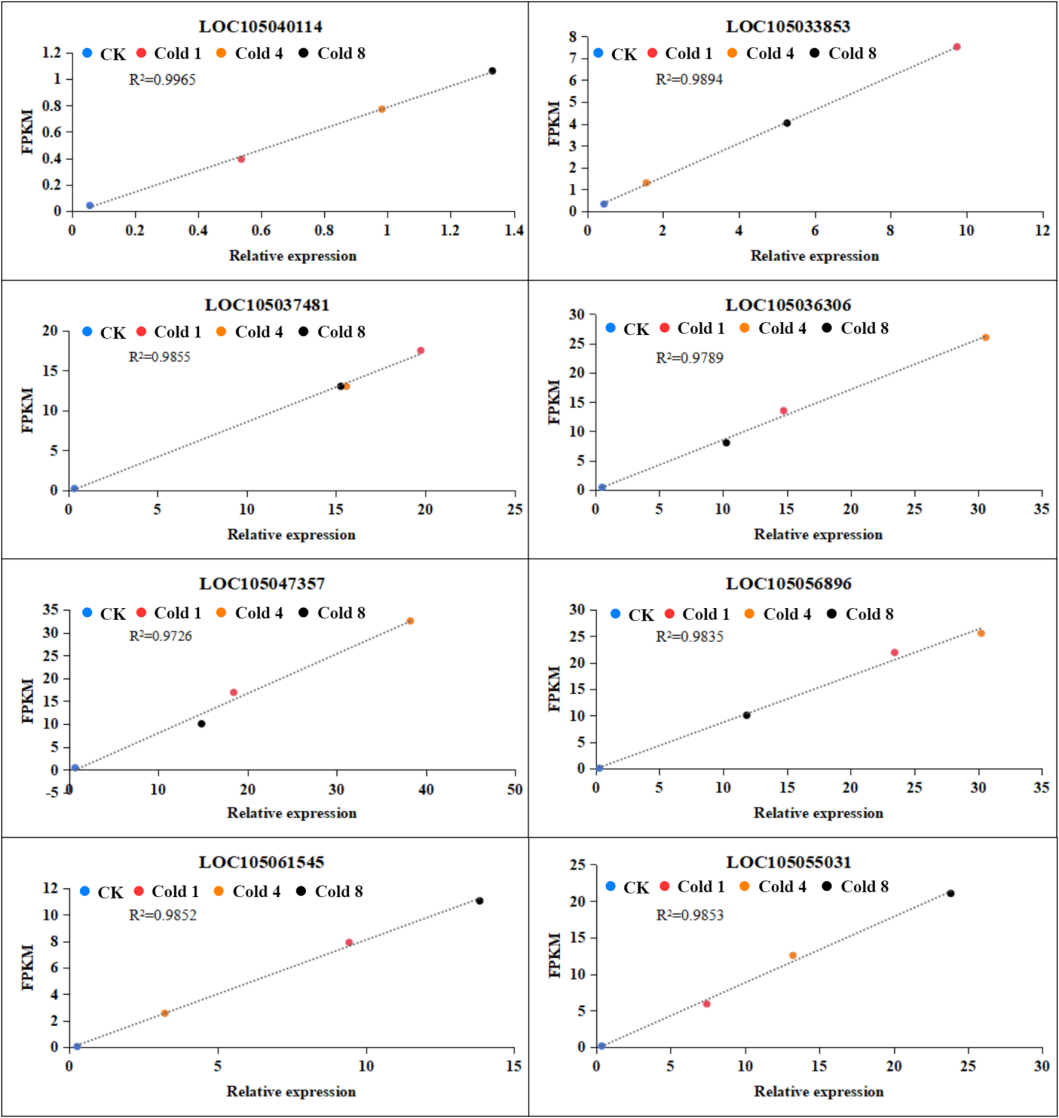
Expression analysis of selected genes under cold stress. Blue dots represent CK, red dots represent Cold 1, orange dots represent Cold 4, and black dots represent Cold 8, data represent mean values ± SD.

The eight differentially expressed genes (DEGs) selected for qRT-PCR validation were chosen based on specific and representative criteria, not randomly, to ensure a rigorous assessment of RNA-Seq reliability. These genes were intentionally selected to span a broad spectrum of fold-change values, in order to evaluate the accuracy of the transcriptome data across varying expression levels. As shown in **Figure 10**, the qRT-PCR results demonstrated high consistency with the RNA-Seq expression trends, strongly supporting the validity of our sequencing data. We are therefore confident that this strategically chosen gene set effectively represents the overall DEG profile and provides robust validation for the RNA-Seq findings.

### 3.9 Hormone signaling and cold adaptation

**Figure 11:**
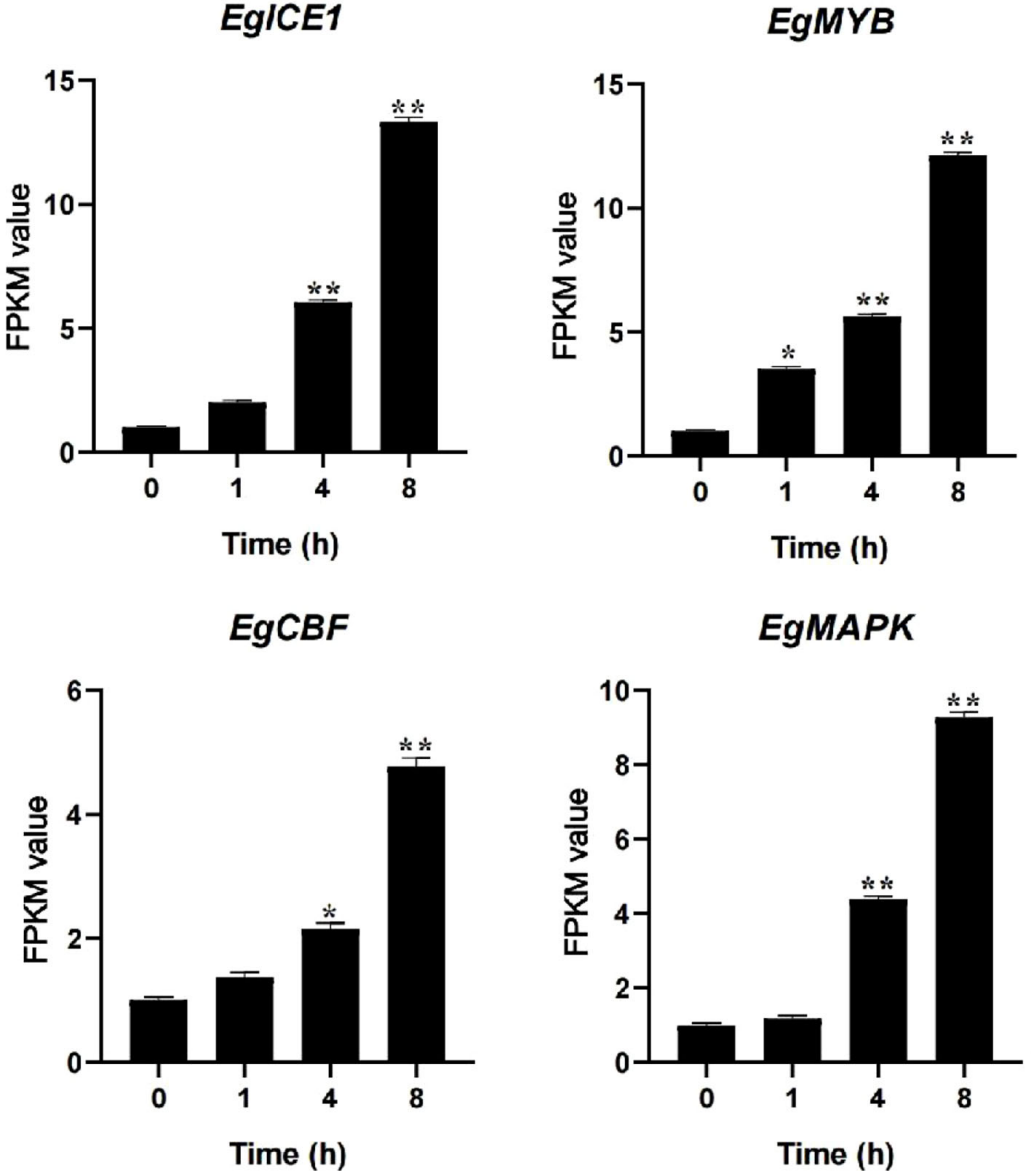
FPKM value of *ICE1*, *MYB*, *CBF* and *MAPK* genes under cold stress conditions. Asterisks represent significant difference at p ≤ 0.05(*) and p ≤ 0.01(**).

The regulatory network governing plant responses to cold stress involves a tightly coordinated interplay between gene expression control, hormonal signaling, and environmental perception. These interconnected mechanisms collectively enable plants to adapt to cold conditions. Phytohormones serve as key mediators, playing a central role in orchestrating physiological and molecular responses to cold stress. In *Arabidopsis*, cold exposure disrupts auxin distribution by impairing the intracellular trafficking of auxin efflux transporters (Shibasaki et al., 2009). Similarly, in tea plants, three R2R3-MYB transcription factors (*CsMYB45*, *CsMYB46*, and *CsMYB105*) respond to cold stress by activating the JA signaling pathway (Han et al., 2022). Additionally, the plasma membrane-localized protein AtCor413pm1 in *Arabidopsis* functions as an ABA-responsive regulator, facilitating cold adaptation through ABA signal transduction (Hu et al., 2021). Further integrating immune and cold responses, the key transcription factor *ICE1* in *Arabidopsis* interacts with the SA signaling pathway to modulate plant immunity during cold stress (Li et al., 2024). In wheat, the SA methyltransferase TaSAMT1 was recently identified, revealing a novel regulatory mechanism where the brassinosteroid (BR) signaling master regulator *TaBZR1* epigenetically modulates SA metabolism (Chu et al., 2024). These findings illustrate the complexity of plant cold stress responses, which integrate hormonal crosstalk, transcriptional regulation, and epigenetic modifications to enhance cold tolerance. Such insights provide a valuable foundation for improving crop cold resistance through genetic engineering or targeted breeding strategies. Specifically, we observed a simultaneous and dramatic induction of *ICE1* (*LOC105051080*) (12.33-fold), *MYB* (*LOC105052782*) (11.08-fold), and *CBF* (*LOC105054150*) (3.76-fold) homologs under cold stress condition for 8 h, highlighting the potential conservation and activation of this key pathway in a tropical woody oil crop. Our finding showed that the *MAPK* homolog (*LOC140856367*) was highly upregulated (8.24-fold) presents an intriguing new angle for oil palm cold tolerance (**Figure 11**). We hypothesize that this induced MAPK may serve as a critical upstream regulator that regulate the ICE1-MYB-CBF cascade in oil palm, potentially through post-translational modification. This represents a novel and species-specific regulatory layer atop the conserved transcriptional circuit. Therefore, our work not only confirms the activation of universal cold-responsive players in oil palm but also delineates a potential species-specific regulatory hierarchy, with MAPK acting as a putative master switch. This discovery provides valuable candidate genes (particularly *MAPK* and *ICE1*) for genetic engineering strategies aimed at enhancing cold tolerance in this critically important, yet cold-sensitive, tropical crop.

#### 3.9.1 Auxin

**Figure 12:**
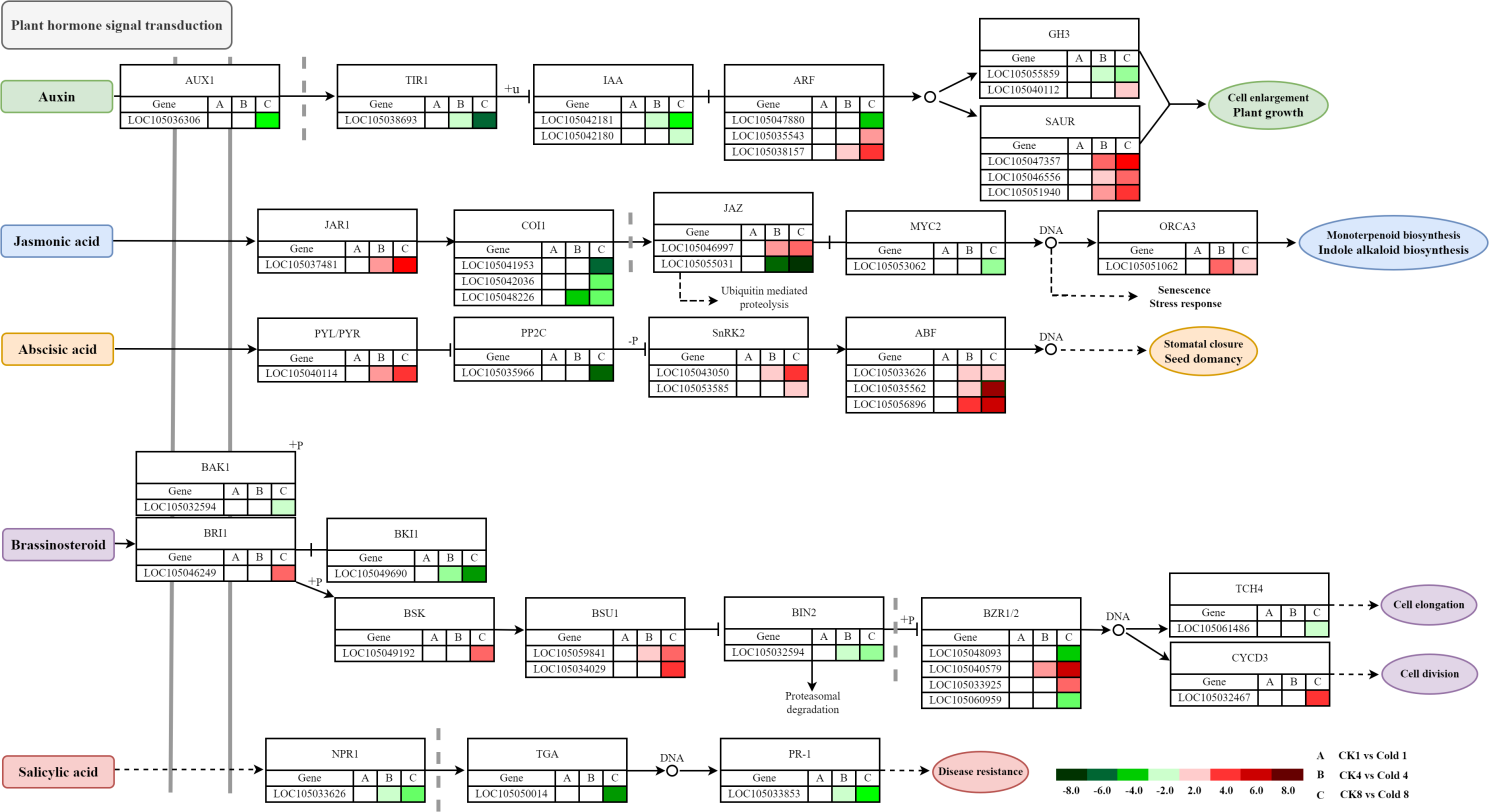
Heatmap visualization of gene expression dynamics in plant hormone signal transduction. Red and green hues denote up- and down-regulated DEGs, respectively, based on FPKM values.

Core components (TIR1/AFB–Aux/IAA–ARF) integrate auxin with ABA and ROS under cold (Rahman, 2013; Yu et al., 2017; Sharma et al., 2021; Du et al., 2022). We identified *AUX1* (*LOC1050536306*), *TIR1* (*LOC105038693*), *IAA* (*LOC105042181*, *LOC105042180*), *ARF* (*LOC105047880*, *LOC105035543*, *LOC105038157*), *GH3* (*LOC105055859*, *LOC105040112*), and *SAUR* (*LOC105047357*, *LOC105046556*, *LOC105051940*) (**Figure 12**). Downregulation of *AUX1*/*TIR1* suggests reduced transport/perception; *SAUR* genes were upregulated, whereas *AUX1*, *TIR1*, *IAA*, *ARF*, and *GH3* were downregulated, indicating auxin pathway suppression to conserve energy.

#### 3.9.2 Jasmonic acid

JA signaling (JAR1–COI1–JAZ–MYC2) coordinates stress responses (Carvalhais et al., 2017; Hu et al., 2017; Huang et al., 2024). Under cold, *JAR1* (LOC105037481) was upregulated, *COI1* (LOC105041953, LOC105042036, and LOC105048226) downregulated, and JAZ genes were differentially expressed (**Figure 12**), suggesting possible COI1-independent activation. Downregulation of *MYC2* may release repression on stress-responsive genes (e.g., *ORCA3*), promoting adaptation.

#### 3.9.3 Abscisic acid

PYR/PYL/RCAR receptors bind ABA to inhibit PP2Cs, releasing SnRK2s, which activate ABF transcription factors (Chen et al., 2020; Kou et al., 2024). Under cold, upregulation of *PYL/PYR* (*LOC105040114*) and downregulation of *PP2C* (*LOC105035966*) activate *SnRK2* (*LOC105043050*, *LOC105053585*) and amplify ABA signaling, increasing ABF (*LOC105033636*, *LOC105035562*, and *LOC105056896*) activity and ABA-responsive gene expression (**Figure 12**), aligning with the classical model (Li et al., 2011). These results identify that oil palm responds to cold stress via a conserved ABA-dependent mechanism. The findings provide molecular evidence for ABA signaling in tropical crops like oil palm and highlight potential targets for cold-tolerance breeding.

#### 3.9.4 Brassinosteroids

BRI1–BAK1 receptor activation initiates signaling through BSK–BSU1–BIN2–BZR1/2, with TCH4 and CYCD3 as effectors (Hafeez et al., 2021; Han et al., 2023; Kim and Russinova, 2020). Increased expression of *BRI1* (*LOC105046249*), *BSK* (*LOC105049192*), *BSU1* (*LOC105059841*, *LOC105034029*), and *CYCD3* (*LOC105032467*), and decreased *BAK1* (*LOC105032594*), *BKI1* (*LOC105049690*), *BIN2* (*LOC105054741*), and *TCH4* (*LOC105061486*) support BR pathway activation with nuanced regulation. BZR1/2-related genes showed mixed regulation, suggesting paralog-specific responses (**Figure 12**).

#### 3.9.5 Salicylic acid

NPR1–TGA–PR1 is central to SA signaling (Chen et al., 2019; Ding et al., 2018). Reduced expression of *NPR1* (*LOC105033626*), *TGA* (*LOC105050014*), and *PR1* (*LOC105033853*) suggests that SA-dependent SAR may not be triggered under cold (**Figure 12**).

### 3.10 MAPK pathway–mediated cold tolerance

**Figure 13:**
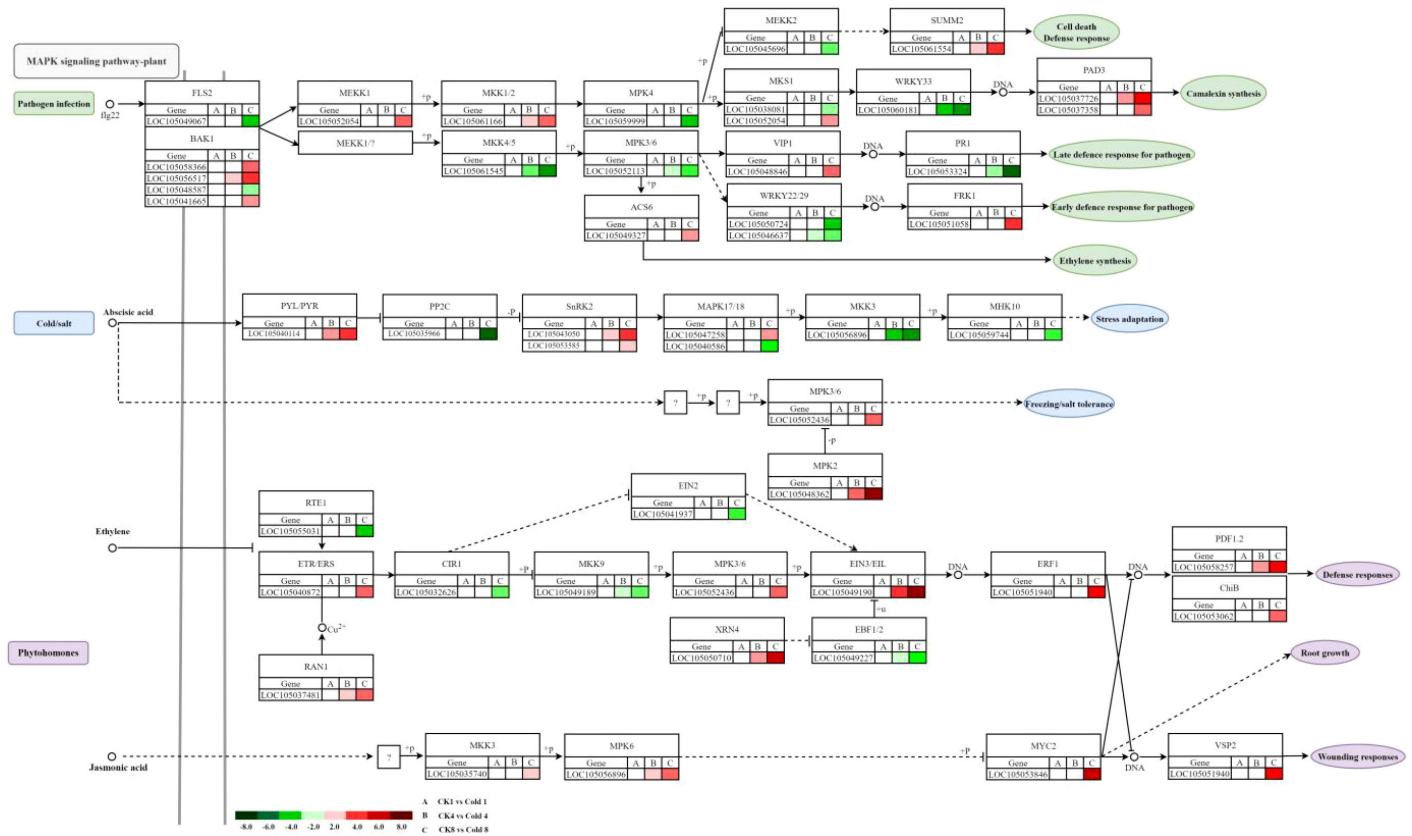
Heatmap visualization of different genes in MAPK signaling pathway. Red and green hues denote up- and downregulated DEGs, respectively, based on FPKM values.

Key cold-responsive genes in the MAPK cascade participate in pathogen defense and ABA/ethylene/JA responses (Iqbal et al., 2022). In pathogen signaling, *FLS2* (*LOC105049067*) was downregulated; *BAK1* genes (*LOC105058366*, *LOC105056517*, *LOC105041665*) were upregulated; *MEKK1* (*LOC105052054*) and *MKK1/2* (*LOC105061166*) were upregulated, while *MKK4*/5 (*LOC105061545*), *MPK4* (*LOC105059999*), and *MPK3/6* (*LOC105052113*) were downregulated, indicating activation of the MEKK1–MKK1/2 branch and suppression of the MEKK2–MKK4/5–MPK3/6/4 branch (**Figure 13**). Ethylene biosynthesis (*ACS6*; *LOC105049327*) was upregulated. *VIP1* (*LOC105048846*) was upregulated, whereas *WRKY22/29* (*LOC105050724*, *LOC105046637*) and *PR1* (*LOC105053324*) were downregulated; *FRK1* (*LOC105051058*) and *PAD3* (*LOC105037726*, *LOC105037358*) were upregulated, suggesting prioritization of JA/ET-mediated rapid defenses over SA-dependent long-term resistance. In ABA signaling, *PYL*/*PYR* (e.g., *LOC105040114*) was upregulated, *PP2C* (*LOC105035966*) downregulated, *SnRK2* (*LOC105043050*, *LOC105053585*) upregulated, and *MAPK17/18* (*LOC105047258*) upregulated. Selective downregulation of *MKK3* (*LOC105056896*) and *MHK10* (*LOC105059744*) suggests pathway-specific modulation. In ethylene signaling, upregulation of *ETR*/*ERS* (*LOC105040872*), *RAN1* (*LOC105037481*), *EIN3/EIL* (LOC105049190), *ERF1* (*LOC105051940*), *PDF1*.2 (*LOC105058257*), and *ChiB* (*LOC105053062*), along with downregulation of *RTE1* (*LOC105055031*) and *EBF1*/*2* (*LOC105049227*), indicates activation of canonical and MAPK-linked branches, with post-transcriptional regulation (*XRN4*; *LOC105050710*). In JA signaling, increased *MKK3*, *MPK6*, *MYC2*, and *VSP2* suggest MAPK-mediated JA activation and JA–ethylene co-regulation via *ERF1* and *PDF1.2. MYC2* and *MPK6* emerge as targets for improving cold resilience, consistent with SlMYC2–SlCBF1/2 regulation in tomato (Li et al., 2025).

## 4 Discussion

### 4.1 Integration of physiological and transcriptomic responses reveals a coordinated cold defense strategy

Cold exposure induces ROS accumulation, causing membrane destabilization and energy dysregulation (Xing et al., 2022). Plants activate antioxidant systems (SOD, POD) to restore redox homeostasis and mitigate damage (**Figures 1A, B**) (Liu et al., 2023; Foyer and Noctor, 2005). Transcriptome results indicated that the expression levels of the oil palm *SOD* gene (*LOC105059069*) and *POD* gene (*LOC105040036*) were up-regulated after 4 hours of cold stress (**Figures 1K, L**). The FPKM values were 5.32-fold and 7.03-fold higher than those in the control group, respectively. The observed increase in antioxidant enzyme activities is underpinned by the transcriptional activation of their corresponding genes, demonstrating a multi-level strategy to combat oxidative burst. Soluble sugars (e.g., sucrose, glucose, fructose) accumulate as compatible osmolytes to stabilize osmotic potential (**Figure 1D**). The expression levels of key sugar-synthesis-related genes, Sucrose Phosphate Synthase (*SPS*, *LOC105036668*) and Sucrose Synthase (*SuS*, *LOC105050124*), were significantly up-regulated after 4 hours of cold stress, with FPKM values 3.88-fold and 6.27-fold higher than those in the control group, respectively (**Figures 1M, N**). The massive accumulation of soluble sugars is not merely a passive consequence but an active process, facilitated by the transcriptional reprogramming of both biosynthetic and transport machinery. Cold increases membrane permeability, accelerates unsaturated fatty acid degradation, promotes lipid peroxidation, elevates MDA, and increases REC (**Figures 1C, E**). Low temperatures suppress key enzymes in chlorophyll biosynthesis, impair chloroplast development, and reduce chlorophyll a/b (**Figures 1F–J**). Transcriptome analysis revealed that the expression of Glutamyl-tRNA reductase (*HEMA*, *LOC105054529*) was down-regulated, with an FPKM value 0.21-fold that of the control group (**Figure 1O**). The cold-induced chlorophyll degradation results from a concerted transcriptional suppression of anabolic pathways and induction of catabolic pathways, effectively dismantling the photosynthetic apparatus to conserve resources and mitigate photo-oxidative damage. CO_2_ assimilation declines as electron transport is disrupted and lower stomatal conductance limits gas exchange (Li et al., 2020), while respiratory ATP production is hindered (Kang et al., 2023), collectively inhibiting growth. The cold-induced chlorophyll degradation results from a concerted transcriptional suppression of anabolic pathways and induction of catabolic pathways, effectively dismantling the photosynthetic apparatus to conserve resources and mitigate photo-oxidative damage.

### 4.2 Transcriptomic signatures of cold stress

The number of differentially expressed genes (DEGs) increased over the course of treatment, peaking at 8 hours post-cold exposure with 6,585 DEGs identified in the CK 8 h vs Cold 8 h comparison (**Figures 2A, B**). Notably, at this time point, the number of down-regulated genes exceeded that of up-regulated genes. This result suggested that this pattern as a central adaptive mechanism employed by plants under low-temperature stress, attributable largely to energy reallocation and extensive transcriptional reprogramming: i) In response to stress, plants suppress a range of energy-demanding basal biological processes, including photosynthesis, cell division, and ribosome biogenesis, to reallocate finite resources toward stress adaptation and survival mechanisms (Liu et al., 2022; Zhang et al., 2020). Consequently, genes associated with these pathways are broadly down-regulated. Our results are consistent with this phenomenon, showing pronounced enrichment and suppression of genes related to photosynthetic and ribosomal pathways. ii) In order to response cold stress, a select set of functionally important genes is up-regulated to enact protective responses, such as the production of osmoregulators (e.g., proline, soluble sugars), antioxidant enzymes (e.g., SOD, POD) (Zhou et al., 2022, 2025). This characteristic transcriptomic profile, marked by widespread down-regulation alongside targeted up-regulation, is well-documented in studies of plant abiotic stress. In conclusion, the prevalence of down-regulated genes at 8h should not be interpreted as a diminished stress response; rather, it reflects a strategic and efficient shift from a growth-oriented state to a defense-oriented state at the transcriptional level. These insights provide valuable temporal resolution for understanding molecular mechanisms underlying cold tolerance in oil palm.

RNA-seq profiling across 0–8 h cold exposure revealed DEGs in abiotic stress adaptation, developmental plasticity, and pathogen defense crosstalk, aligning with findings in other species (Li et al., 2024; Zhang et al., 2023; Peng et al., 2023). Enriched BP terms included cellular activity, metabolism, biological regulation, regulation of biological processes, and localization (**Figure 3**), indicating reorganization of energy distribution, activation of regulatory networks (e.g., transcription factors), and maintenance of cellular structures (Zhu, 2016; He et al., 2024; Kim et al., 2024). At 8 h, BP terms “metabolic process”, “cellular process”, and “response to stimulus” were prominent (**Figure 4A**), consistent with metabolic reprogramming, secondary metabolite synthesis, and homeostatic regulation (Wu et al., 2023; Hotamisligil and Davis, 2016). MF terms (catalytic activity, binding) reflected roles for enzymes and binding proteins (e.g., calmodulin, transcription factors) in stress adaptation (Jan et al., 2023), while CC terms implicated structural remodeling (Hu et al., 2024). KEGG analysis highlighted activation of plant–pathogen interactions, MAPK signaling, and hormone signaling (**Figure 6**). Under cold, MAPK components (e.g., *MPK3/6*) regulate transcription factors (*ICE1*, *MYB*/*CBF*) to modulate cold-responsive genes (Wen et al., 2023). ABA biosynthesis/signaling (PYR/PYL–PP2C–SnRK2) induces stomatal closure and stress gene expression (Li et al., 2023); ethylene via EIN3/EIL affects cell elongation and antifreeze proteins (Su et al., 2024); JA and SA bridge cold and immune signaling (Wang et al., 2020; Wu et al., 2019); auxin shifts reflect growth–defense trade-offs (Liu et al., 2024).

This study reveals, for the first time in oil palm, a differential regulatory phenomenon within the MAPK cascade, marked by pronounced activation of the MEKK1–MKK1/2 branch and concurrent suppression of the MEKK2–MKK4/5–MPK3/6/4 branch (Zhou et al., 2023). This branch-specific pattern may underlie a key cold adaptation strategy in oil palm as a tropical crop (Ye et al., 2017). Transcriptomic analyses further demonstrate that oil palm downregulates salicylic acid (SA) pathway markers (*WRKY22/29*, *PR1*) while upregulating jasmonic acid/ethylene (JA/ET)-mediated defense markers (*FRK1*, *PAD3*, *PDF1.2*), indicating a distinct preference in defense signaling compared to model plants (Bi et al., 2021; Kovács et al., 2025). This reprogramming may reflect a specialized mechanism for coping with low-temperature stress. The study further delineates key nodes of crosstalk between MAPK and hormone signaling pathways, including: i) The central role of the MPK6–MYC2 module in activating JA signaling. ii) The specific involvement of MAPK17/18 in ABA signal transduction. iii) A post-transcriptional regulatory mechanism mediated by the EIN3–EBF1 node within ethylene signaling. Together, these findings construct a unique MAPK–hormone regulatory network in oil palm. It is confirmed that *MYC2* and *MPK6* occupy central positions in this network, exhibiting both evolutionary conservation and species-specific features when compared to the SlMYC2–SlCBF1/2 regulatory axis in tomato. These results provide concrete targets for molecular breeding aimed at enhancing cold resilience in oil palm. These insights not only clarify the architecture of cold stress signaling in oil palm but also offer novel theoretical foundations and genetic resources for improving cold adaptation in tropical crops.

### 4.3 Prospects for molecular breeding

The findings elucidate the precise molecular mechanisms underlying oil palm’s response to cold stress, providing a clear roadmap for molecular breeding and biotechnological intervention. In this context, the widespread downregulation of genes in growth-promoting pathways (e.g., auxin, brassinosteroid) is not a passive impairment but an active, energy-saving strategy. By shutting down non-essential metabolic processes, the plant reallocates precious resources to activate and sustain critical defense responses, primarily orchestrated by ABA. This reprogramming ensures survival at the expense of temporary growth arrest. Our data, showing coordinated downregulation across multiple pathways, provide a comprehensive transcriptomic portrait of this conserved ecological trade-off phenomenon. The genes we identified as downregulated in auxin, JA, and BR pathways represent prime candidates for knockdown or CRISPR/Cas-mediated gene silencing in crops (Maharajan et al., 2022). Creating alleles with reduced expression of these genes could genetically predispose plants to a “primed” state, constitutively allocating more resources and thus enhancing hardness (Ma et al., 2024). The development of molecular markers associated with key genes significantly related to cold resistance, such as the superior allelic variations of *LOC105059069* (*SOD*) and *LOC105040036* (*POD*), can be used to screen breeding materials with enhanced ROS-scavenging capacity (Wang et al., 2025). Constructing expression vectors containing SOD or POD genes driven by stress-inducible promoters and transforming them into oil palm enables targeted and efficient ROS clearance, avoiding potential growth penalties associated with constitutive overexpression. Overexpressing core positive regulators in the JA signaling pathway, or using gene editing technologies (e.g., CRISPR/Cas9) to knock out negative regulators in the ABA pathway, can remodel the hormone network to favor cold resistance activation. Enhancing the expression or activity of positive regulatory branches in the MAPK cascade amplifies the cold warning signal, prompting earlier and stronger activation of downstream defense mechanisms. These strategies break the limitations of the natural gene pool, creating novel or rare superior alleles that enable leapfrog improvements in cold tolerance. Given that cold tolerance is a complex trait controlled by multiple genes, the effect of modifying a single gene may be limited. These findings suggest a strategy for synergistic multi-gene manipulation.

Compared to advances in cold tolerance research on tropical crops like cassava and banana, oil palm research can benefit from establishing a well-defined evaluation framework based on physiological indicators, antioxidant profiles, and osmolyte accumulation to enable precise assessment of cold tolerance across diverse germplasms (Santanoo et al., 2022; Wei et al., 2022). Both cassava and banana boast large germplasm collections, with core sets of cold-tolerant accessions already identified. Likewise, a systematic screening of oil palm genetic resources should be carried out to build a curated cold-tolerant core collection, serving as a critical source of parental material for conventional hybrid breeding. Moreover, a comparative biology strategy should be actively employed (Xu et al., 2022; Li et al., 2022). Oil palm research can efficiently target and adapt key pathways, substantially reducing redundant exploratory work and accelerating progress. Together, these approaches will deliver a robust scientific foundation for developing new cold-resilient oil palm varieties and strengthening agricultural sustainability.

## 5 Conclusions

Cold stress triggers significant physiological and molecular responses in oil palm, including enhanced antioxidant activity and the accumulation of soluble sugars and MDA. Transcriptomic analysis revealed time-dependent regulation of DEGs, with notable enrichment in hormone signaling (IAA, JA, ABA, BR) and the MAPK cascade. Together, these pathways orchestrate cold adaptation through extensive transcriptional reprogramming, predominantly characterized by the downregulation of energy-costly processes, coupled with the upregulation of specific protective mechanisms. These findings, and especially the identification of key genes within these regulated pathways, offer a foundation for targeted breeding and genetic engineering strategies aimed at enhancing oil palm resilience.

## Data Availability

The data presented in this study are publicly available. The data can be found here in the China National Gene Bank Database (https://db.cngb.org/cnsa) under accession numbers CNP0006920 and CNP0007547.
